# Gait Recognition with Self-Supervised Learning of Gait Features Based on Vision Transformers

**DOI:** 10.3390/s22197140

**Published:** 2022-09-21

**Authors:** Domagoj Pinčić, Diego Sušanj, Kristijan Lenac

**Affiliations:** Faculty of Engineering, University of Rijeka, Vukovarska 58, 51000 Rijeka, Croatia

**Keywords:** gait recognition, self-supervised learning, Gait Energy Image (GEI), people identification, vision transformers

## Abstract

Gait is a unique biometric trait with several useful properties. It can be recognized remotely and without the cooperation of the individual, with low-resolution cameras, and it is difficult to obscure. Therefore, it is suitable for crime investigation, surveillance, and access control. Existing approaches for gait recognition generally belong to the supervised learning domain, where all samples in the dataset are annotated. In the real world, annotation is often expensive and time-consuming. Moreover, convolutional neural networks (CNNs) have dominated the field of gait recognition for many years and have been extensively researched, while other recent methods such as vision transformer (ViT) remain unexplored. In this manuscript, we propose a self-supervised learning (SSL) approach for pretraining the feature extractor using the DINO model to automatically learn useful gait features with the vision transformer architecture. The feature extractor is then used for extracting gait features on which the fully connected neural network classifier is trained using the supervised approach. Experiments on CASIA-B and OU-MVLP gait datasets show the effectiveness of the proposed approach.

## 1. Introduction

Gait is a biometric feature that describes the walking pattern of every individual. Compared with other biometric features such as the face, iris, fingerprint, and ears, gait has several unique properties. Gait can be captured from a greater distance than face or iris, which also means that the person does not have to interact with the sensor, i.e., a camera. In addition, gait is difficult to change, making it a reliable biometric feature. The gait of an individual can also be extracted from low-resolution sensors, such as those found in most current surveillance cameras. The range of applications of gait biometric is wide, e.g., in surveillance scenarios, access control, and identification of individuals for crime investigation purposes.

Gait biometric has several limitations when applied in the real world. First, factors such as illumination changes, shadows, and occlusions can significantly alter the appearance of an individual’s gait. Second, the cameras that capture an individual’s gait often have different viewing angles, resulting in drastically different appearances of the gait, even though the individual’s gait signature is the same. Third, the carrying modalities are also commonly present, such as individuals wearing a bag, coat, hat, or another accessory, which visually change the individual’s gait from an appearance perspective.

In the literature, there are two general approaches for tackling the task of gait recognition. The first is compressing the silhouettes of a single gait cycle of an individual into a single image, which serves as a gait features representation [1,2]. Han et al. [1] propose compressing the individual’s binary silhouettes of one gait cycle, extracted from video frames by background subtraction, into one compact gait representation, called Gait Energy Image (GEI). The second approach considers the gait as a sequence of silhouettes of an individual, which are used individually as input for feature extractor [3,4,5]. In both approaches, the current state-of-the-art methods rely exclusively on deep learning. From their emergence in 2012 [6], CNNs have in recent years dominated the field of image-based deep learning, and have naturally become the standard backbone network used in approaches tackling gait recognition [3,4,7,8,9]. Wu et al. [7] extracted gait features using the deep CNNs via similarity learning. GaitSet [3] proposed treating the silhouettes of an individual as a set using custom CNN with triplet loss for gait representation learning. Rijun et al. [5] extracted gait features using the information about an individual’s pose throughout video frames, using the CNN for predicting the body pose from an image. Bai et al. [10] addressed the problem of radar-based gait recognition based on the dual-channel CNN. Chen et al. [11] used CNN network for gait classification based on the multistatic micro-doppler signatures. A detailed description of a typical gait identification pipeline can be found in [3,7,12].

However, in recent years, a new architecture emerged as a direct competitor to CNNs in the field of image classification—vision transformers (ViTs). ViT architecture was proposed by Dosovitskiy et al. [13], applying the standard transformer encoder from the field of natural language processing to the field of computer vision, i.e., image classification task. ViTs have shown excellent results on many image classification benchmarks [13,14,15], demonstrating their strong generalization capability. Compared with CNNs, ViTs demand fewer computational resources to train and have stronger modeling capability. Still, their application in the domain of gait recognition has not yet been explored.

All of the previously mentioned methods use a supervised approach to address the gait recognition problem. Supervised deep learning requires that annotated samples are available for training and test data, which can be expensive to obtain. Moreover, many of the state-of-the-art methods use complex model architectures to extract useful gait features [3,4,8,16]. Complex deep learning models often result in long training time, slow convergence, and a large number of model parameters that need to be tuned.

In this manuscript, we propose a new architecture and learning approach for gait recognition. Since labeling all samples in a dataset is an expensive and time-consuming process, we propose using a self-supervised approach for learning useful gait features from the input data. The self-supervised approach has emerged in recent years [17,18,19,20] and has been successfully applied to a number of problems [21,22]. The main goal of self-supervised learning is to learn useful data representations from the unlabeled data by creating a pretext task. The pretext task involves predicting an occluded portion of an input image based on the rest of the image. In this manuscript, we opted to use the DINO [20] approach. DINO showed excellent results on the image classification dataset ImageNet and outperformed previous self-supervised approaches based on CNNs at a significantly lower computational cost. This approach uses the ViT model as a backbone, which has an interesting property compared with CNNs trained in the same way. It has been shown that the self-supervised approach in conjunction with ViTs results in ViTs models learning to separate the desired object from the background without explicit guidance [20]. GEI images are used as input data for the DINO model, representing a single gait cycle of an individual.

Our approach uses the general ViT architecture as a backbone model, in conjunction with DINO self-supervised learning method, for learning useful gait features from GEI images of individuals, which can then be used as input to a simple fully connected neural network (FCNN) classifier to classify individuals.

## 2. Related Work

### 2.1. Gait Recognition

Gait recognition methods can generally be divided into two categories: image-based and video-based methods.

Image-based methods extract individual’s silhouettes from a video sequence through background subtraction, and then align and compress them into a single image that represents the final gait representation [1,2]. Gait features are then extracted from the images either using Principal component analysis (PCA) [1], Linear discriminant analysis (LDA) [2,23,24], or CNN [25,26,27]. Finally, the similarity between features is computed, for example, by using the cosine similarity. Some methods also propose to integrate the above steps into an end-to-end network [28]. Wang et al. [29] proposed the feature-distribution-consistent Generative Adversarial Networks (GAN) to tackle the problem of cross-view gait recognition.

Video-based methods extract silhouettes from a video sequence similar to image-based methods; however, instead of compressing them into a single representation, they are used in their raw form as input data. For every individual, all silhouettes are fed into the network and gait features are extracted. Chao et al. [3] proposed a method called GaitSet, which considers the gait as a set consisting of permutable silhouettes. The method is able to learn identity information from the set and proved to be effective in solving the problem of different viewpoints and different carrying conditions. GaitPart [4] improved the aforementioned method by considering that the different body parts of an individual carry information of different significance, and thus modeled the different spatio-temporal representations for different body parts. mmGaitSet [30] is another improvement of the GaitSet method, in which information about an individual’s body posture was incorporated into the network. Some approaches propose to use a model-based gait feature based on the individual’s body pose to solve the problem [5,31,32]. Wolf et al. [33] proposed the use of 3D convolutions to better capture the spatio-temporal features of gait. Although video-based methods produce better results than image-based methods, they are generally more difficult to train.

Regarding the type of backbone network used in the mentioned deep learning approaches, the CNN’s are used almost exclusively.

### 2.2. Self-Supervised Learning

In recent years, a new learning paradigm in deep learning has piqued the interest of researchers—self-supervised learning (SSL). Self-supervised learning aims at solving the ever-present problem of the lack of data for training deep learning models. By using self-supervision, the model learns without any labels, by means of pretext learning, where one part of the input data is learned from another part of the same input. Many self-supervised methods exist nowadays, such as [17,18,19,20,34]. SimCLR [18] used contrastive learning, with contrastive loss function, by maximizing the similarity between two augmented views of the same image. BYOL [17] used two networks, online and target network, which have the same architecture but different weights. The target network trains the online network, and the target network’s weights are updated through the exponential moving average of the online network. SwAV [34] used instance-level discrimination, where each image or its transformation is considered as a separate class. The goal of the approach is to learn an embedding in a way that semantically similar images are grouped closer together in the features space, through means of using contrastive loss and image augmentation.

In DINO [20], the knowledge distillation with no labels is used. The DINO framework consists of two networks—teacher $\Phi_{t}$ and student $\Phi_{s}$—that share the same architecture but different parameters, $\phi_{t}$ and $\phi_{s}$, respectively. The goal of the student network is to match the probability distribution of the teacher network. The method uses a multicrop strategy [34] during training, where for every input image two global views are generated (about 50% of the input image), along with several local views of the same image (less than 50% of the input image). The global views pass through the teacher network while the global and local views both pass through the student network. The cross-entropy loss is used to measure similarity between output vectors from the teacher and student network. The student parameters $\phi_{s}$ are learned by minimizing the cross-entropy loss with stochastic gradient descent, while the teacher parameters $\phi_{t}$ are defined as an exponential moving average of the student parameters. In that way, the framework is able to gradually learn useful features from input images, learning the global to local correspondences from different views of the same image. Further, in contrast to many SSL methods [18,19], DINO does not require negative samples, which greatly simplifies the training procedure.

### 2.3. Self-Supervised Gait Recognition

Self-supervised deep learning approaches have only recently attracted the interest of researchers in the area of gait recognition. As a result, there are not many methods that use SSL for gait recognition. Among the first to investigate the application of SSL learning to gait recognition was WildGait [35]. In their manuscript, the authors created the novel Uncooperative Wild Gait (UWG) dataset, in which gait representations are automatically annotated by recognizing skeletal sequences of individuals. In addition, they propose the use of the SSL approach for pretraining the Spatio-Temporal Graph Convolutional Network to utilize a large number of samples for creating useful gait representations. Finally, the model is fine-tuned in a supervised manner to the target datasets and evaluated. Another SSL approach to the gait recognition task is SelfGait [36]. In the aforementioned manuscript, the authors propose using the SSL approach for learning the spatio-temporal gait representation from unlabeled samples. They use the horizontal pyramid mapping (HPM) [3] and micro-motion template builder (MTB) [4] spatio-temporal backbones, which are specifically designed for the gait recognition task. As in WildGait [35], the proposed approaches use CNN as the backbone network.

## 3. The Proposed Approach

**Figure 1:**
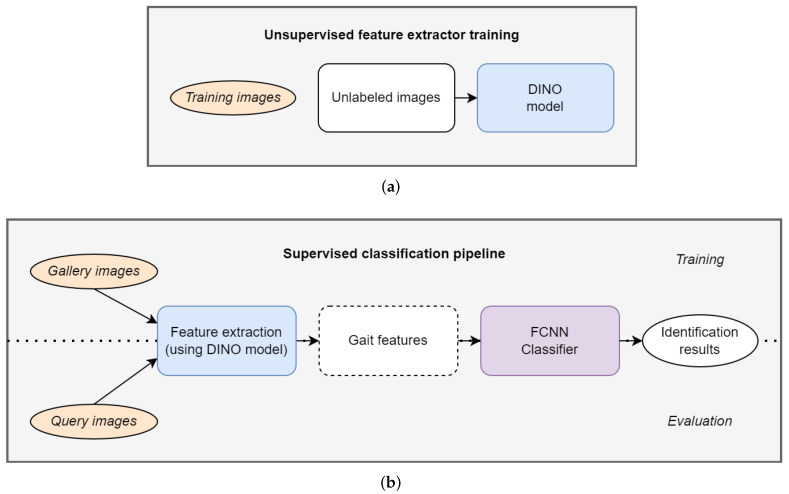
Gait recognition pipeline. (**a**) Training feature extractor. (**b**) Classification pipeline.

In this section, we describe our proposed approach, along with a detailed explanation of its key components. The overall processing pipeline is depicted in Figure 1. The first part of our proposed approach uses the DINO self-supervised model to learn gait features from unlabeled training data, as shown in Figure 1a. Next, a simple FCNN is used as a classifier for the features obtained by the DINO feature extractor model, and is trained on gallery samples and tested on query samples, as shown in Figure 1b. Labeled samples are only needed for training the FCNN classifier, as the classifier is trained using a supervised approach.

### 3.1. Preprocessing

The first step in our proposed approach is data preparation. In general, assuming the input data are in the form of raw RGB image sequences taken from a camera, the typical gait data preprocessing steps [12,27] are applied. First, the noise is filtered from the images. Second, the silhouettes are extracted for every subject in binary form, using, e.g., background subtraction method. Third, images are normalized so that all silhouettes have the same height and are horizontally aligned. Then, a gait cycle estimation is performed in order to construct a final gait representation. In this manuscript, image-based gait features are used in the form of GEI [1]. GEI is able to preserve the static information of a gait sequence, such as the shape of the subject’s body, and the subject’s dynamic information, such as the variation of frequency and phase during the subject’s locomotion. The GEI representation *G* for a given gait cycle can be calculated with the formula

(1) $$G\left(i,j\right)=\frac{1}{N}\sum_{t=1}^{N}I\left(i,j,t\right),$$

where *N* represents the number of silhouette frames in the gait cycle, *t* represents the frame number in a gait cycle at a moment in time, and I(i,j) is the original silhouette image with (i,j) values in the 2D image coordinate.

### 3.2. Learning Discriminative Gait Features

**Figure 2:**
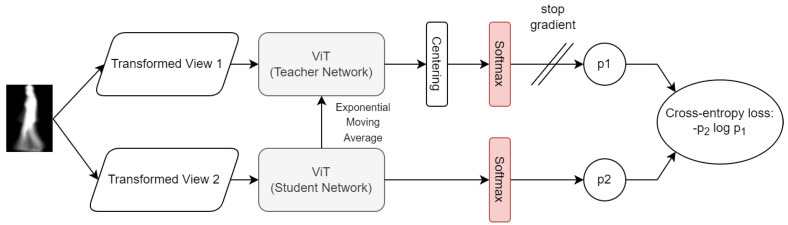
DINO self-supervised learning [20]. The goal of the student network is to match the probability distribution of a teacher network using cross-entropy loss, given different views of the same input image.

The second step in our proposed approach is training the feature extractor. In this manuscript, we propose using a self-supervised learning paradigm in order to tackle the problem of learning discriminative gait features. We use the recently proposed method called DINO [20], which showed promising results in various computer vision tasks such as image classification and image retrieval. The DINO architecture is depicted in Figure 2.

Originally, DINO constructs a set of eight local views (96×96 crops, passed only through $\Phi_{s}$) and two global views (224×224 crops, passed through both $\Phi_{t}$ and $\Phi_{s}$). In this work, to adapt to gait-specific data, we use eight local views but with local crops of size 20×20, while two global crops are of size 64×64. We change crop sizes in order to adapt to the sizes of our gait training images while retaining the similar ratios of global and local crops as in the original manuscript. Moreover, since the DINO was originally trained on ImageNet, we change the augmentations used during training, by removing most of the image augmentations used (color jitter, Gaussian blur, solarization, random horizontal flip) and using only the random erasing augmentation, since the aforementioned augmentations do not bring a performance gain when used on gait-specific data.

The DINO method exhibits the ability to segment the foreground objects in an image, i.e., object boundaries, in a self-supervised manner. In natural images, such as ImageNet, foreground object segmentation is a difficult problem, considering that many possible variations of the foreground object and the background exist. In a gait recognition scenario, where images are presented in the form of, e.g., GEI, the foreground object, i.e., a subject, is clearly outlined in relation to the background, which could lead to the model focusing its attention on the most significant parts of an image such as the dynamic features presented as pixels in the range of 〈0,255〉.

Since gait datasets lack the large amount of data needed to train the ViT model from scratch [13], the fine-tune strategy is used in this work. The DINO model is trained on the ImageNet dataset and then fine-tuned to gait data.

We propose using the DINO method as a feature extractor to produce discriminative features of input images to be used later for classification.

### 3.3. Vision Transformers

The DINO uses the vision transformer model [13] as its backbone network, although CNN’s also work without modifying the general DINO architecture. The ViTs input consists of patches of resolution p×p that represent non-overlapping sections of the input image. For an image *I*,

(2) $$I\in R^{H\times W\times C},$$

where *H* represents the height of an image, *W* represents its width, and *C* is the number of channels in an image, the resulting image patches are

(3) $$I\in R^{N\times p^{2}C},$$

where $N=\frac{HW}{p^{2}}$ is the number of patches and *p* is the patch resolution.

Patches are linearly projected into an embedding, and a CLS token is added, which serves as a class token, i.e., representation of the entire input image, and is used for the actual classification. Furthermore, at this step, the positional embeddings are added to help the model retain the positional information of input patches. Then, patch embeddings, positional embeddings, and CLS token are passed through the standard Transformer Encoder, which consists of self-attention and feed-forward layers, with skip connections. Finally, the output CLS token of the Transformer Encoder is sent to a Multilayer Perceptron (MLP) model for classification.

We use the small ViT model, as defined by Touvron et al. [37]. Furthermore, we train models with a patch size of 16 and 8 to investigate the influence of patch size on model performance.

### 3.4. Classifier

**Figure 3:**
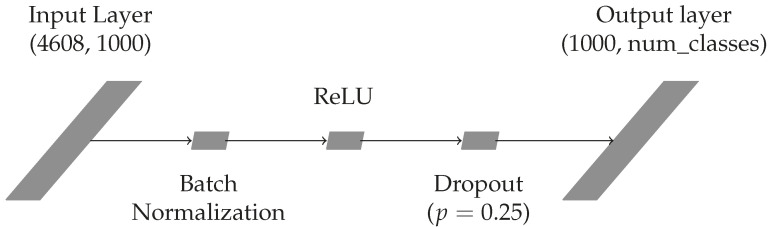
Proposed FCNN classifier.

After the DINO feature extractor model is trained, the gait features for gallery and query image can be extracted and used for classification. In order to classify the features, we propose using a simple FCNN classifier. Accordingly, we set the gait recognition problem as a gait classification problem, where the gallery acts as training data for the FCNN classifier and query acts as test data. For example, if a gallery contains 100 subjects we consider that a classification problem with 100 classes. We design a simple FCNN—depicted in Figure 3—that consists of two linear layers, together with batch normalization, ReLU activation function, and dropout. The hyperparameters of a proposed FCNN are determined empirically. Additionally, we use the center loss [38] to further facilitate learning a more diverse feature representation. The main loss used is the cross-entropy loss, and the combination with center loss is given by the formula

(4) $$L=L_{ce}+\alpha L_{c},$$

where *L* represents final loss value; $L_{ce}$ and $L_{c}$ are values of cross-entropy loss and center loss functions, respectively; and α is a scalar that balances influence of the center loss on the overall loss value and is set to α=0.0001.

As in feature extractor training, the images were normalized according to the custom dataset’s normalization values. Random erasing is used as a data augmentation technique. Furthermore, in order to boost representation learning, we concatenate the CLS tokens from all 12 blocks of the DINO model as a final input image representation that serves as input to the FCNN classifier. Dimensionality of CLS token for the small ViT model is 384; thus, the input dimensionality of FCNN classifier is 4608.

## 4. Experimental Setup

To validate the proposed approach, we conducted experiments to assess the performance of the proposed DINO feature extractor model and the performance of the FCNN classifier trained on features extracted with the feature extractor model. Experiments were conducted in a way that allows for easy comparison with current state-of-the-art models used in gait recognition, following the same dataset splits and comparison metrics. The experimental setup is described next; then, the results are presented and analyzed.

### 4.1. Datasets

In this manuscript, we conducted experiments on two widely used gait recognition datasets: CASIA-B [39] and OU-MVLP [40], where CASIA-B a presents a smaller but widely used dataset, while OU-MVLP presents one of the largest gait datasets to date. The aforementioned allows for analyzing the performance of the proposed approach on a smaller or larger dataset, to see if the data amount is critical in training a successful DINO feature extractor.

CASIA-B dataset [39] is one of the most popular gait datasets in the literature. It consists of 124 subjects, three different walking conditions, and 11 different views (0–$180^{\circ }$ with an increment of $18^{\circ }$). Walking conditions are normal (NM) with six sequences per subject, walking with a bag (BG) with two sequences per subject, and walking with a coat or a jacket (CL) also with two sequences per subject. In total, 110 sequences are available for each subject in the dataset. Since in this manuscript we use GEI images, the aforementioned translates to almost 13,600 images in total, with an average of 110 images per subject. We conduct experiments on three partition settings for training and testing, commonly used in literature. First, the ST (small-sample) setting uses the first 24 subjects for training and the rest (100 subjects) are used for testing. Second, the MT (medium-sample) setting uses the first 62 subjects for training and the rest (62 subjects) are used for testing. Third, the LT (large-sample) setting uses the first 74 subjects for training and the rest (50 subjects) are used for testing. In all three partition settings, the first 4 sequences of the NM modality are used in the gallery, while the remaining 6 sequences of NM modality are used in the query along with the 2 sequences of BG and CL modalities.

OU-MVLP dataset [40] is one of the largest public gait datasets available today. It consists of 10,307 subjects and 14 different views ($0^{\circ }$–$90^{\circ }$ and $180^{\circ }$–$270^{\circ }$, in increments of $15^{\circ }$) per subject. For every view, there are two sequences (#00–01). For training, 5153 subjects are used, while for testing, the rest of the 5154 subjects are used. In the test set, sequences with index #01 are used as a gallery, while the ones with index #00 are used as a query. In total, there are over 267,000 GEI images, with approximately 26 GEI images per subject.

Additionally, we resize all images from both datasets to size 64 × 44 as performed in [3,36], to ensure comparison compatibility as well as lowering computing requirements for training the DINO model. Furthermore, when training the DINO model, the training data are normalized using the mean and stdev calculated from the used training data.

### 4.2. Experiments

In order to evaluate the performance of our proposed approach, we constructed GEI image representations for each subject in each dataset. Then, we trained DINO feature extraction models on two aforementioned datasets, CASIA-B and OU-MVLP. For each dataset, two models were trained: one with a patch size of 16 and one with a patch size of 8. Next, a simple FCNN classifier was trained on gallery samples, to construct the final model for gait classification. Finally, the trained FCNN classifier was evaluated using the query samples.

### 4.3. DINO Implementation Details

For the implementation of the DINO method, the official GitHub repository was used [41], with slight modifications, as explained in Section 3.2, to account for the different data distribution of gait data in comparison with natural images of ImageNet dataset, such as adjusted global and local crop sizes and different training data augmentations. In order to fine-tune both the student and the teacher networks, the full ImageNet pretrained DINO model checkpoint was used. In our experiments, we used only small ViT models, which roughly correspond to the size of normal Resnet-50 [42] architecture by the number of parameters in the network. We trained models with patch sizes 16 and 8 to study the effect of patch size on the model’s accuracy. The remaining DINO model parameters, such as momentum teacher value, teacher temperature, and global and local crop scales are the same as in the original manuscript [20].

### 4.4. Training Details

We trained the DINO models for 1000 epochs, with a batch size of 32 for all experiments on the CASIA-B and OU-MVLP datasets. The optimizer used was AdamW [43] with a learning rate of 0.0005. The training was performed using one Nvidia 2080Ti 11 GB GPU.

The FCNN classifier was trained for 100 epochs, with a batch size of 128. The Adam optimizer was used for FCNN classifier with a learning rate of 0.0005; similarly, the Adam was used for the center loss optimizer with a learning rate of 0.1.

For both DINO models and the FCNN classifier, the learning rates were determined empirically. The learning rates were searched within the range of 0.1 to 0.000001 using the grid search method. The number of epochs for training the DINO model was set to 1000, as the accuracy did not improve when training the model for longer. Similarly, the number of epochs for training the FCNN classifier was set to 100. The batch size for both models was set by finding the optimal value between the batch sizes of 8 and 128, with steps of the power of 2.

### 4.5. Evaluation Protocol

For evaluation of our experimental results, we use rank-1 accuracy, where we look at the percentage of predictions where the top prediction is the correct one, i.e., matches the ground-truth value. Additionally, the identical-view cases are excluded for comparability with other state-of-the-art methods.

## 5. Results

In this section, the results of conducted experiments are presented. It is worth noting that, except SelfGait [36], which uses self-supervised learning, every other method compared uses a supervised learning approach. Furthermore, the state-of-the-art methods mentioned in this section use silhouettes as input data, as well as features extracted directly from frames of a subject walking, while the method proposed by Liao et al. [27] uses GEIs, the same as our method.

### 5.1. CASIA-B

For the ST setting, the results are presented in Table 1. Compared with the other state-of-the-art methods, our method achieves the highest accuracy in the NM and BG modality. Although, the CL modality accuracy is the lowest among the state-of-the-art methods.

In the MT setting, Table 2, the overall accuracy of the NM modality of our method outperforms the rest of the methods again, while the BG modality is below the rest of the methods. Further, the CL modality showed significantly lower results.

Finally, in the LT setting, Table 3, our method again gained the best accuracy in the NM modality, while the BG modality is comparable although lower in accuracy than the rest of the methods. CL modality in this setting showed poor accuracy.

Overall, our approach performs best on the NM modality, regardless of the CASIA-B dataset setting. The BG modality performs best in the ST setting, and in the other settings, it is comparable with other methods. The CL modality showed the lowest accuracy in all the settings. The reason for that could be that our model focused its attention primarily on the NM modality, which has the most training data and is easiest to discriminate, without any other covariate condition. BG modality considers the subject carrying a bag, which alters the subject’s appearance slightly; thus, the results for BG modality are overall comparable with those of other state-of-the-art methods. The CL modality considers the subject wearing a coat, which alters the subject’s appearance significantly; as a result, it is the hardest modality available in the dataset, on which our method achieved low accuracy. As such, our proposed method on CL modality may not be the best choice in practical applications, compared with other methods. Further research into boosting the proposed method’s accuracy in the mentioned modality will be performed. Considering the presented results, our approach showed the ability to perform well across different modalities, excluding CL modality. Furthermore, our method discriminates well across the different angles of subjects at which they are recorded. The best accuracy is obtained for the angles that are closer to values of $0^{\circ }$ and $180^{\circ }$, while the lowest are in the area around the $90^{\circ }$ angle.

Both models with patch size 16 and patch size 8 performed similarly in the NM modality, without significant differences in accuracy, across all dataset settings. The significant differences in accuracy arise in BG and CL modalities, where the model with patch size 8 showed significant improvement in accuracy compared with the model with patch size 16. This effect could be due to the ability of the model with patch size 8 to focus its attention to smaller parts of the image, hence, building a model that is more robust to the effect of covariate factors such as a bag or a coat.

### 5.2. OU-MVLP

In Table 4, the results for the OU-MVLP dataset are presented. The results show that our approach achieved comparable results with the other state-of-the-art methods. Our method performs well across all angles—specifically, the $210^{\circ }$ and $225^{\circ }$ angles—while the lowest accuracy is at an angle of $180^{\circ }$. The method SelfGait [36] also uses the self-supervised learning approach but with a specialized backbone network that enhances the spatio-temporal ability of the model, and it achieves the state-of-the-art result on this dataset. In contrast, our approach uses a standard unmodified ViT network, with simple FCNN as a classifier, and achieves comparable accuracy. As the OU-MVLP dataset contains many images, the DINO model was able to learn discriminative features and achieve results comparable with the state-of-the-art. Compared with SelfGait, the advantage of our approach is that it uses a simple general ViT architecture, as opposed to the gait-specific network used in SelfGait. In addition, our method does not explicitly infer temporal features from the data, unlike SelfGait, which uses MTB to learn temporal features from silhouettes, thus making our method more straightforward in terms of learning since only appearance features are learned.

The model with patch size 16 performed slightly better on this dataset compared with the model with patch size 8. As, in this dataset, there are no covariate conditions such as a bag or coat, the model with patch size 8 does not bring any performance improvement as in CASIA-B dataset.

### 5.3. Self-Attention Visualization

In order to assess the features learned by the DINO model, we visualize the different attention heads in the last multihead self-attention block. A random image from each of the datasets is chosen, for which the attention is displayed. The model used was the ViT small model, which has n=6 heads per self-attention block.

**Figure 4:**
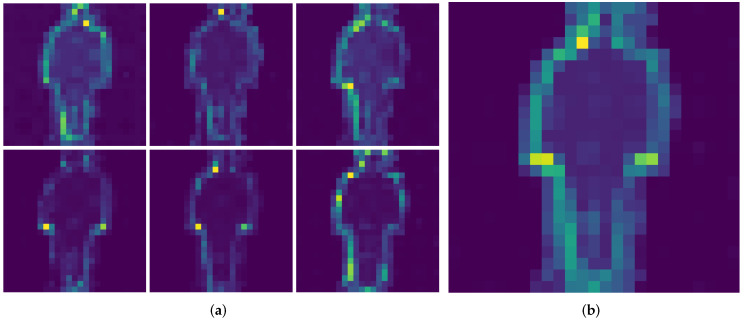
Self-attention of the CLS token on random CASIA-B sample image. (**a**) Self-attention heads. (**b**) Average of all self-attention heads.

**Figure 5:**
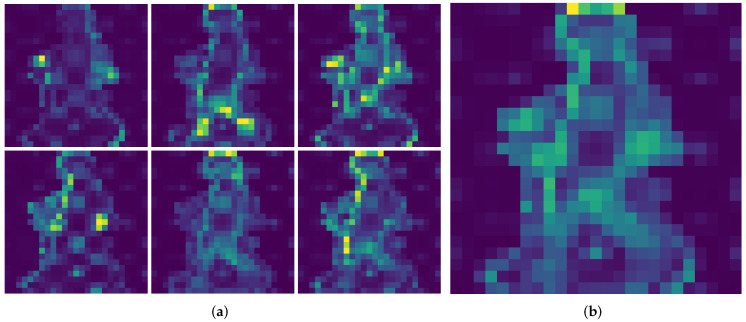
Self-attention of the CLS token on random OU-MVLP sample image. (**a**) Self-attention heads. (**b**) Average of all self-attention heads.

In Figure 4 and Figure 5, the random images from CASIA-B and OU-MVLP datasets are shown, respectively. As depicted in Figure 4a and Figure 5a, each head learns different features from the data, as its attention is focused on different parts of the image. Some attention heads are focused on the subject’s head, while others are on the legs or the left or right part of the subject in the image. Figure 4b and Figure 5b show the average of all attentions across all the heads. This observation is consistent with the ones from the original DINO manuscript, where it is noted that the DINO method successfully segments objects of interest inside the image. In GEI images, the most important area of the image is the outline of the subject, which our proposed approach successfully detects and uses that information for the classification of subjects, producing good results, as shown in Section 5.

### 5.4. Ablation Experiments

In this section, the effectiveness of the vision transformer backbone network and the proposed classifier is studied.

To evaluate the effectiveness of the vision transformer network, we trained the DINO model with the Resnet-50 as a backbone network for comparison. The Resnet-50 is chosen because it has a similar number of parameters in the network compared with the small ViT network, with 23 million and 21 million parameters, respectively. Both models were trained on the CASIA-B dataset’s LT setting, for 1000 epochs and with a patch size of 16. Hyperparameters of the small ViT model were determined as described in Section 4.4, while for the Resnet-50 model the same methodology was used, setting the lr=0.005. In both models, the full ImageNet pretrained DINO model checkpoint was used for fine-tuning. For evaluation, the FCNN network proposed in Section 3.4 was used. In Table 5, the comparison of accuracy of Resnet-50 and the small ViT model is shown. It is evident that the small ViT model significantly outperforms the Resnet-50 model in accuracy across all modalities, proving the effectiveness of the ViT model for the problem of gait recognition.

In Table 6, the comparison of different classifiers is shown. To study the effectiveness of the proposed FCNN classifier, we evaluated the trained ViT feature extractor model using the standard weighted nearest neighbors classifier (k-NN) as in [46]. The feature extractor model used was the small ViT model with a patch size of 16. The FCNN classifier is the same as proposed in Section 3.4. An evaluation is performed on the CASIA-B dataset using the LT setting. The results show that the proposed FCNN classifier significantly outperformed the k-NN classifier in all modalities and angles, especially in the BG modality.

## 6. Conclusions

In this manuscript, we propose a novel approach that uses self-supervised learning for application in the gait recognition task. Using the DINO self-supervised method, the useful gait features are learned using training samples without any annotations. The obtained model is used as a feature extractor for gallery and query images. The simple FCNN classifier is trained using the features extracted from gallery images, and query images are evaluated using the trained model. Experiments conducted on two widely used gait recognition datasets, CASIA-B and OU-MVLP, showed that our proposed approach achieved good results, outperforming the supervised approaches in some cases. Moreover, the self-supervised feature extractor focused its attention on the outlines of the individuals in the GEI images, deeming the outline as the most meaningful information in the image. Taking into account covariate factors, such as different camera viewpoints and different carrying modalities, our method also produced good results comparable with those of other state-of-the-art methods, considering both supervised and self-supervised approaches. We also note that our approach is one of the first that employs ViTs in the domain of gait recognition. In future work, we will investigate the effect of training the feature extractor on specific parts of an image such as the legs, torso, or head on recognition accuracy. Furthermore, additional work will be conducted to further reduce the gap between poorer BG and CL modality results compared with those of NM modality in CASIA-B dataset. Newly proposed variants of vision transformers will also be tested in conjunction with DINO to further boost the recognition accuracy.

## Figures and Tables

**Table 1:** Results for CASIA-B dataset ST setting. The best results for each angle and overall are in bold.

Gallery (NM #1–4)		**0–180°**											**Mean**
Query		$0^{\circ }$	$18^{\circ }$	$36^{\circ }$	$54^{\circ }$	$72^{\circ }$	$90^{\circ }$	$108^{\circ }$	$126^{\circ }$	$144^{\circ }$	$162^{\circ }$	$180^{\circ }$	
NM (#5–6)	GaitSet [3]	64.60	83.30	90.40	86.50	80.20	75.50	80.30	86.00	87.10	81.40	59.60	79.54
	mmGaitSet [30]	78.50	90.70	94.00	92.20	88.10	84.40	87.40	91.70	92.40	90.60	73.90	87.63
	Huang et al. [44]	67.40	81.60	88.80	87.00	80.70	74.90	79.20	86.70	88.20	82.00	66.70	80.29
	Lima et al. [31]	62.50	97.90	87.50	64.60	93.80	95.80	93.80	97.90	70.80	91.70	75.00	84.66
	Proposed ViTs16	99.50	100.00	99.50	100.00	100.00	100.00	99.50	100.00	100.00	99.50	99.50	99.77
	Proposed ViTs8	100.00	100.00	100.00	100.00	99.00	98.50	99.00	100.00	100.00	100.00	100.00	99.68
BG (#1–2)	GaitSet [3]	55.80	70.50	76.90	75.50	69.70	63.40	68.00	75.80	76.20	70.70	52.50	68.64
	mmGaitSet [30]	70.40	81.40	84.70	82.70	77.40	73.00	77.90	83.00	82.00	79.60	65.40	77.95
	Huang et al. [44]	57.80	70.60	77.10	76.20	70.10	64.30	68.70	76.00	75.40	70.30	54.60	69.19
	Lima et al. [31]	52.10	70.80	58.30	43.80	79.20	81.20	77.10	77.10	66.70	77.10	52.10	66.86
	Proposed ViTs16	78.50	68.50	64.50	50.51	54.00	59.00	60.50	58.79	60.80	70.05	72.36	63.41
	Proposed ViTs8	87.00	86.00	74.50	71.72	69.50	69.00	75.00	70.85	74.87	90.36	91.96	78.25
CL (#1–2)	GaitSet [3]	29.40	43.10	49.50	48.70	42.30	40.30	44.90	47.40	43.00	35.70	25.60	40.90
	mmGaitSet [30]	42.20	54.60	58.30	57.00	53.00	49.50	51.40	52.20	51.20	45.60	34.40	49.95
	Huang et al. [44]	33.40	47.10	53.10	48.80	46.10	41.20	47.40	47.70	47.10	39.00	29.30	43.65
	Lima et al. [31]	22.90	29.20	35.40	33.30	39.60	62.50	52.10	52.10	33.30	43.80	33.30	39.77
	Proposed ViTs16	20.50	25.50	18.50	16.08	17.00	16.00	14.50	15.08	17.59	19.80	20.50	18.28
	Proposed ViTs8	30.00	32.50	34.50	27.14	25.50	33.00	25.00	18.59	21.11	26.40	30.00	27.61

**Table 2:** Results for CASIA-B dataset MT setting. The best results for each angle and overall are in bold.

Gallery (NM #1–4)		0–180°											Mean
Query		$0^{\circ }$	$18^{\circ }$	$36^{\circ }$	$54^{\circ }$	$72^{\circ }$	$90^{\circ }$	$108^{\circ }$	$126^{\circ }$	$144^{\circ }$	$162^{\circ }$	$180^{\circ }$	
NM (#5–6)	GaitSet [3]	86.80	95.20	98.00	94.50	91.50	89.10	91.10	95.00	97.40	93.70	80.20	92.05
	mmGaitSet [30]	94.40	98.10	99.30	98.20	96.10	94.40	96.30	98.10	98.40	97.90	92.30	96.68
	Huang et al. [44]	86.70	95.40	97.80	96.30	91.60	87.00	91.40	96.80	95.90	93.30	82.50	92.25
	Liao et al. [27]	63.10	79.40	84.60	79.80	77.00	72.60	77.40	80.30	84.00	78.50	63.70	76.40
	Proposed ViTs16	100.00	100.00	100.00	100.00	100.00	100.00	99.19	100.00	100.00	100.00	100.00	99.93
	Proposed ViTs8	99.19	100.00	100.00	100.00	99.19	99.19	99.19	100.00	100.00	100.00	99.19	99.63
BG (#1–2)	GaitSet [3]	79.90	89.80	91.20	86.70	81.60	76.70	81.00	88.20	90.30	88.50	73.00	84.26
	mmGaitSet [30]	90.50	95.00	94.30	94.60	91.60	88.90	91.20	93.90	94.90	92.30	84.80	92.00
	Huang et al. [44]	80.10	89.90	91.30	87.80	84.00	75.80	81.10	88.60	90.70	85.50	73.70	84.41
	Liao et al. [27]	47.50	59.60	64.20	66.30	61.30	56.70	63.40	63.30	61.80	57.50	47.00	58.96
	Proposed ViTs16	83.06	86.29	75.81	73.98	66.94	63.71	68.55	68.55	73.39	82.11	85.48	75.26
	Proposed ViTs8	88.71	92.74	87.10	81.30	77.42	70.16	77.42	80.65	75.00	88.62	90.32	82.68
CL (#1–2)	GaitSet [3]	52.00	66.00	72.80	69.30	63.10	61.20	63.50	66.50	67.50	60.00	45.90	62.53
	mmGaitSet [30]	73.60	79.50	82.70	82.20	76.40	73.50	74.70	78.30	77.00	72.60	65.50	76.00
	Huang et al. [44]	58.30	71.10	76.80	71.50	64.50	58.90	64.00	68.50	68.80	59.50	49.10	64.64
	Liao et al. [27]	30.20	43.30	43.40	43.10	43.60	41.90	40.00	40.30	41.40	38.70	29.90	39.62
	Proposed ViTs16	17.74	25.00	23.39	27.42	29.03	23.39	20.97	20.16	21.77	23.39	20.16	22.95
	Proposed ViTs8	24.19	25.00	27.42	23.39	28.23	29.03	25.00	19.35	23.39	24.19	27.42	25.15

**Table 3:** Results for CASIA-B dataset LT setting. The best results for each angle and overall are in bold.

Gallery (NM #1–4)		0–180°											Mean
Query		$0^{\circ }$	$18^{\circ }$	$36^{\circ }$	$54^{\circ }$	$72^{\circ }$	$90^{\circ }$	$108^{\circ }$	$126^{\circ }$	$144^{\circ }$	$162^{\circ }$	$180^{\circ }$	
NM (#5–6)	GaitSet [3]	90.80	97.90	99.40	96.90	93.60	91.70	95.00	97.80	98.90	96.80	85.80	94.96
	mmGaitSet [30]	95.60	99.50	99.90	98.80	95.90	95.40	96.20	98.90	98.90	98.40	94.40	97.45
	Huang et al. [44]	91.10	97.90	99.60	97.30	94.30	91.90	94.90	98.10	98.80	96.20	86.60	95.15
	GaitPart [4]	94.10	98.60	99.30	98.50	94.00	92.30	95.90	98.40	99.20	97.80	90.40	96.23
	Proposed ViTs16	100.00	100.00	100.00	100.00	99.00	99.00	99.00	100.00	100.00	100.00	99.00	99.64
	Proposed ViTs8	99.00	100.00	100.00	100.00	99.00	98.00	99.00	100.00	100.00	100.00	99.00	99.45
BG (#1–2)	GaitSet [3]	83.80	91.20	91.80	88.80	83.30	81.00	84.10	90.00	92.20	94.40	79.00	87.24
	mmGaitSet [30]	91.40	95.60	94.10	94.30	91.40	88.60	90.00	93.00	95.70	95.70	88.10	92.54
	Huang et al. [44]	84.30	91.20	93.40	91.80	86.10	80.30	84.40	90.90	93.70	90.80	80.10	87.91
	GaitPart [4]	89.10	94.80	96.70	95.10	88.30	94.90	89.00	93.50	96.10	93.80	85.80	92.46
	Proposed ViTs16	85.00	86.00	80.00	76.77	72.00	78.00	78.00	77.00	77.00	80.81	84.00	79.51
	Proposed ViTs8	90.00	88.00	90.00	84.85	81.00	80.00	81.00	78.00	79.00	88.89	91.00	84.70
CL (#1–2)	GaitSet [3]	61.40	75.40	80.70	77.30	72.10	70.10	71.50	73.50	73.50	68.40	50.00	70.35
	mmGaitSet [30]	77.60	84.40	85.80	84.70	78.90	76.60	78.50	79.30	82.20	82.80	72.20	80.27
	Huang et al. [44]	64.70	79.40	84.10	80.40	73.70	72.30	75.00	78.50	77.90	71.20	57.00	74.02
	GaitPart [4]	70.70	85.50	86.90	83.30	77.10	72.50	76.90	82.20	83.80	80.20	66.50	78.69
	Proposed ViTs16	20.00	28.00	28.00	28.00	26.00	26.00	24.00	19.00	24.00	27.00	19.00	24.45
	Proposed ViTs8	27.00	31.00	28.00	30.00	40.00	34.00	28.00	24.00	22.00	27.00	32.00	29.36

**Table 4:** Results for OU-MVLP dataset. The best results for each angle and overall are in bold.

Gallery	All 14 Views														Mean
Query	$0^{\circ }$	$15^{\circ }$	$30^{\circ }$	$45^{\circ }$	$60^{\circ }$	$75^{\circ }$	$90^{\circ }$	$180^{\circ }$	$195^{\circ }$	$210^{\circ }$	$225^{\circ }$	$240^{\circ }$	$255^{\circ }$	$270^{\circ }$	
GEINet [25]	11.40	29.10	41.50	45.50	39.50	41.80	38.90	14.90	33.10	43.20	45.60	39.40	40.50	36.30	35.76
Zhang et al. [45]	56.20	73.70	81.40	82.00	78.40	78.00	76.50	60.20	72.00	79.80	80.20	76.70	76.30	73.90	74.66
Zhang et al. [8]	74.00	88.30	94.60	95.40	88.00	91.30	90.00	76.70	89.50	95.00	94.90	88.00	90.80	89.80	89.02
GaitSet [3]	79.50	87.90	89.90	90.20	88.10	88.70	87.80	81.70	86.70	89.00	89.30	87.20	87.80	86.20	87.14
SelfGait [36]	85.10	89.30	92.00	94.30	89.10	90.20	90.90	87.40	91.80	89.30	88.70	90.80	91.60	87.70	89.87
Proposed ViTs16	78.00	88.06	91.11	90.69	86.80	87.69	85.86	83.20	90.57	92.24	91.88	87.10	88.32	86.36	87.71
Proposed ViTs8	75.34	86.14	90.12	88.36	84.99	87.16	85.62	81.09	89.42	90.95	90.29	86.05	88.56	86.38	86.46

**Table 5:** Comparison of Resnet-50 and small ViT model accuracy on CASIA-B dataset using LT setting. The best results for each angle and overall are in bold.

Gallery (NM #1–4)		0–180°											Mean
Query		$0^{\circ }$	$18^{\circ }$	$36^{\circ }$	$54^{\circ }$	$72^{\circ }$	$90^{\circ }$	$108^{\circ }$	$126^{\circ }$	$144^{\circ }$	$162^{\circ }$	$180^{\circ }$	
NM (#5–6)	ResNet-50	80.00	82.00	88.00	78.00	79.00	84.00	81.00	84.00	84.00	83.00	70.00	81.18
	Proposed ViTs16	100.00	100.00	100.00	100.00	99.00	99.00	99.00	100.00	100.00	100.00	99.00	99.64
BG (#1–2)	ResNet-50	60.00	58.00	36.00	29.29	32.00	34.00	28.00	41.00	42.00	50.51	44.00	41.35
	Proposed ViTs16	85.00	86.00	80.00	76.77	72.00	78.00	78.00	77.00	77.00	80.81	84.00	79.51
CL (#1–2)	ResNet-50	18.00	19.00	20.00	16.00	18.00	17.00	6.00	14.00	20.00	24.00	15.00	17.00
	Proposed ViTs16	20.00	28.00	28.00	28.00	26.00	26.00	24.00	19.00	24.00	27.00	19.00	24.45

**Table 6:** Comparison of FCNN and k-NN classifiers accuracy on CASIA-B dataset using LT setting. The best results for each angle and overall are in bold.

Gallery (NM #1–4)		0–180°											Mean
Query		$0^{\circ }$	$18^{\circ }$	$36^{\circ }$	$54^{\circ }$	$72^{\circ }$	$90^{\circ }$	$108^{\circ }$	$126^{\circ }$	$144^{\circ }$	$162^{\circ }$	$180^{\circ }$	
NM (#5–6)	k-NN	48.00	81.00	93.00	81.00	95.00	81.00	87.00	91.00	81.00	79.00	54.00	79.18
	Proposed FCNN	100.00	100.00	100.00	100.00	99.00	99.00	99.00	100.00	100.00	100.00	99.00	99.64
BG (#1–2)	k-NN	38.00	55.00	51.00	43.43	57.00	48.00	46.00	54.00	41.00	48.48	33.00	46.81
	Proposed FCNN	85.00	86.00	80.00	76.77	72.00	78.00	78.00	77.00	77.00	80.81	84.00	79.51
CL (#1–2)	k-NN	8.00	10.00	14.00	16.00	15.00	11.00	11.00	14.00	14.00	18.00	9.00	12.73
	Proposed FCNN	20.00	28.00	28.00	28.00	26.00	26.00	24.00	19.00	24.00	27.00	19.00	24.45

## Data Availability

Not applicable.

## Abbreviations

The following abbreviations are used in this manuscript:

CNN	Convolutional Neural Network
ViT	Vision Transformer
SSL	Self-Supervised Learning
GEI	Gait Energy Image
FCNN	Fully Connected Neural Network
k-NN	k-Nearest Neighbors Algorithm
PCA	Principal Component Analysis
LDA	Linear Discriminant Analysis
GAN	Generative Adversarial Network
UWG	Uncooperative Wild Gait dataset
HPM	Horizontal Pyramid Mapping
MTB	Micro-motion Template Builder
MLP	Multilayer Perceptron
NM	Normal walking condition
BG	Walking with a bag
CL	Walking with a coat or a jacket
ST	Small-sample
MT	Medium-sample
LT	Large-sample
